# Does anxiety influence the use of complementary or alternative medicine among cancer patients?

**DOI:** 10.1007/s00432-025-06173-2

**Published:** 2025-03-26

**Authors:** Caroline Heyder, Judith Büntzel, Hristo Boyadzhiev, Petra Stegmaier, Bijan Zomorodbakhsch, Klaus Heißner, Christoph Stoll, Ludwig Fischer von Weikersthal, Jana Czekay, Ivonne Rudolph, Jutta Hübner

**Affiliations:** 1 Hämatologie Und Internistische Onkologie, Klinik Für Innere Medizin II, Universitätsklinikum Jena, Friedrich-Schiller-Universität Jena, Am Klinikum 1, 07747 Jena, Germany; 2 Klinik Für Hämatologie Und Medizinische Onkologie, Universitätsklinikum Göttingen, Robert-Koch-Straße 40, 37075 Göttingen, Germany; 3 Habichtswald Reha-Klinik, Wigandstraße 3a, 34131 Kassel, Germany; 4 Zentrum Für Strahlentherapie, Wirthstraße 11C, 79110 Freiburg, Germany; 5 Onkologische Kooperation Harz, Kösliner Straße 14, 38642 Goslar, Germany; 6 MVZ Für Innere Medizin, Hämatologie und Onkologie Weiden, Moslohstraße 53, 92637 Weiden, Germany; 7 Klinik Für Innere Medizin, Kulmbacher Straße 103, 95445 Bayreuth, Germany; 8 MVZ St. Marien, Mariahilfbergweg 7, 92224 Amberg, Germany; 9 Onkologisches Forum Celle E.V, Fritzenwiese 117, 29221 Celle, Germany; 10 Waldburg-Zeil Kliniken, Rehabilitationsklinik Bad Salzelmen, Badepark 5, 39218 Schönebeck, Germany

**Keywords:** Complementary and alternative medicine, Anxiety, Trait anxiety, Cancer, CAM methods, Use rate

## Abstract

**Purpose:**

The aim of this study was to investigate the extent to which patients' anxiety due to their cancerous disease has an influence on the use of complementary and alternative medicine (CAM) methods.

**Methods:**

230 patients completed an anonymous voluntary questionnaire that was sent to outpatient oncological facilities participating in the survey. This questionnaire included standardised tests such as the Allgemeine Selbstwirksamkeit Kurzskala (ASKU, self-efficacy short scale) and the State-Trait-Anxiety-Inventory (STAI) as well as socio-demographic information and a section on CAM use. Statistical analyses and regression models were used to identify correlations.

**Results:**

Female gender, high level of education (high school diploma or university degree) and increased trait anxiety were related to CAM use. All other variables analysed showed no significant results.

**Conclusion:**

This study demonstrates that trait anxiety and sociodemographic factors significantly influence CAM usage among cancer patients. Physicians and health care providers should consider this in consultations to guarantee the best possible care for patients.

## Introduction

CAM methods are often used by cancer patients alongside conventional therapy (Huebner et al. 2014a, 2014b; Micke et al. 2009). Previous results showed that socio-demographic factors, such as high education and female gender, but also spirituality and attentiveness are predictors for the use of certain CAM methods (Alsharif 2021; Dufter et al. 2021; Keene et al. 2019; Micke et al. 2009; Truant et al. 2013; Verhoef et al. 2005) as well as high external locus of control (Ebel et al. 2015). According to Huebner et al. 2022, patients with higher self-efficacy show more interest in CAM methods, but there was no evidence of increased use of CAM methods in these patients.

Research also demonstrated that mistrust in conventional medicine was not the main reason for CAM, but that non-physician practitioners and naturopaths take more time for patients (Huebner et al. 2014a). An analysis from 2006 and 2007 in the United States showed that the average visit duration of cancer patients in outpatient physician offices was 22.9 min (Guy et al. 2012). A German study even reports that the initial oncological consultation only lasts 15 min on average (Oskay-Ozcelik et al. 2007). Moreover, the unmet needs of cancer patients primarily include emotional and psychological support (Hart et al. 2022; Wang et al. 2018), such as appropriate listening and encouragement from other family members and professionals, because they are often affected by sadness, loneliness, fear and helplessness (Wang et al. 2018). In addition, the German Cancer Society (Leitlinienprogramm Onkologie 2023) reported that the international prevalence of affective disorders among cancer patients is 13.0%, and that of anxiety disorders 11.1%, which is consistent with other sources (Caruso et al. 2020, Fernando et al. 2023).

Based on the results of the studies conducted so far, which examined some influencing factors and patient characteristics related to CAM use, we wanted to know whether there is a relationship between anxiety of oncological patients and CAM use and found no previous studies in this regard.

Therefore, we hypothesised that the prevalence of CAM use in cancer patients with anxiety might be higher than in cancer patients without anxiety, as CAM methods might serve as a coping mechanism to reduce cancer-related anxiety.

## Methods

### Study design

This multicentre cross-sectional study took place in seven German oncology centres from August 2023 to December 2023. No pilot study was carried out in advance.

### Study participants

The survey was aimed at patients with cancer who visited one of the seven German oncology centres to which the questionnaires were sent by post or electronically during the survey period. These oncology centres included three oncological medical care centres, two rehabilitation clinics, a radiotherapy practice and a cancer counselling centre. The participating centres were asked to print out a sufficient number of questionnaires and display them in their facilities during the survey period. In most cases, the questionnaires were given to patients at registration or were available in the waiting rooms. Patients were asked to take part in the study anonymously by completing the questionnaires themselves and returning them to the medical staff. Study participants were able to choose the location where they wanted to complete the questionnaire to guarantee their privacy. The oncological facilities returned the complete questionnaires to us by post or as scan per email.

As many patients as possible should be reached in a low-threshold manner. An online-only survey would have excluded patients who are less tech-savvy from participating, e.g. older people, as cancer is a disease of old age. We therefore opted for an analogue survey.

The prerequisites for participation were that the patients were at least 18 years old at the time of the survey and had sufficient knowledge of the German language to answer the questions. The participants were informed that the survey was anonymous and voluntary and were asked to complete the printed questionnaires by hand.

### Questionnaire

By completing the questionnaire, the patients agreed to participate in the anonymous and voluntary survey. The questionnaire consisted of four different parts and a total of 47 questions or statements that had to be evaluated.

Next to (1) demographic data, (2) a validated, reliable and objective tool (Beierlein et al. 2014) for assessing self-efficacy, the ASKU (Allgemeine Selbstwirksamkeit Kurzskala, Beierlein et al. 2014) was used, before (3) patients were asked to check if they used any of the CAM method listed by the questionnaire. There was the option of ticking "yes" or "no" for each method and a field for free text information to specify any herbal substances used.

Then (4) we assessed patients' anxiety using the German short version of the State-Trait-Anxiety-Inventory (STAI) (Grimm 2009). The latter is a reliable psychological test that has been pretested by the developers (Laux et al. 1981) and measures state anxiety and anxiety as a trait of the participants. It is based on the German version of the STAI (Laux et al. 1981) and consists of two independent scales for self-description, each with 10 descriptions of feelings. Using an eight-point Likert scale, participants were able to select whether and how often these statements applied to them. In the STAI there were negative statements as well as positive ones. So, the patients had to read the statements carefully to put the crosses correctly when expressing their feelings. In retrospect, the positive assertions were recoded in the evaluation, so that a low STAI-value expresses low anxiety and vice versa (Grimm 2009). This validated test suited our target group perfectly, as it is written in German and is also relatively short, which increased the chance that patients would complete the questionnaire during their waiting time in oncological practices.

### Statistical analysis

Data collection and statistical analyses were conducted in IBM SPSS Statistics Version 29. The ASKU was evaluated according to the user manual (Beierlein et al. 2014) as well as the STAI (Grimm 2009; Laux et al. 1981). The ASKU consisted of three questions. Patients could tick how much they agreed with each statement on a five-point Likert scale. Average values were calculated from the three answers and compared with the reference values from the manual (Beierlein et al. 2014). The possible results of the average values were between one and five. Values of one correspond to the lowest possible self-efficacy. Values of five correspond to the highest possible self-efficacy (Beierlein et al. 2014).

The STAI consisted of ten statements on state anxiety and ten statements on trait anxiety (Grimm 2009). The patients were able to tick a box on a scale of eight levels to indicate how much they agreed with the statements (Grimm 2009). At the end, the total values were calculated separately for state anxiety and trait anxiety. The possible results of the total scores were between 20 and 80 points (Laux et al. 1981). Sum values of 20 points showed the lowest possible state- or trait-anxiety. Sum values of 80 points showed the highest possible state or trait anxiety. Sum values of our interviewees were compared with reference values from the manual STAI (Laux et al. 1981).

A binary logistic regression analysis was carried out to objectify the influence of different variables on the use of CAM. The dependent dichotomous variable was the use of CAM methods. The independent variables were divided into three blocks (block 1: gender, age, level of education, religion, block 2: anxiety and depression as previous illnesses, mean value of the state anxiety inventory, mean value of the trait anxiety inventory, block 3: mean value of the ASKU). To determine the model quality of our binary logistic regression analysis, we calculated the significance using Chi-square (Backhaus et al. 2006) and the explained variance using Nagelkerke's R^2^ (Nagelkerke 1991). The latter can assume a value from zero to one. The closer this value is to one, the greater the effect size of the test. In addition, the correlation between STAI and ASKU was calculated according to Bravais-Pearson. p-values < 0.05 were considered as significant.

### Ethical vote

All data were taken anonymously. The ethical approval was authorized by the ethics committee at the university hospital of the Friedrich-Schiller-Universität Jena (2021–2130-Bef). The survey was conducted on a voluntary basis and had no influence on the further treatment of the patients. The respondents took part in the study without payment and consented to anonymous data analysis by completing the questionnaire.

## Results

### Demographics and general information

A total of 230 people took part in the survey (Table 1). Some participants did not fully complete the questionnaire; this was accounted for in the analyses. 159 participants were female (69.4%), 69 were male (30.1%) male and one person (0.4%) did not specify their gender. The age of the surveyed ranged from 18 to 90 years, the average age at the time of the interview was 62 years (mean value = 61.57 years, median = 63.00 years, standard deviation = 12.25, minimum = 18 years, maximum = 90 years). Due to the skewness (skewness = −0.466) and the kurtosis (kurtosis = 0.288), the age in our survey of cancer patients did not correspond to a normal distribution. This was in line with our expectations due to the increased prevalence of cancer with increasing age. In the following analyses, we counted high school diploma (13.0%) and university degree (17.0%) as the highest level of education and differentiated this from all the other qualifications. Patients could tick whether they were Catholic, Protestant, Orthodox, Muslim, Jewish, atheist or belonged to another religion or did not wish to make a statement in this regard. In the evaluation, however, we then summarised the religions and only looked at whether the patients were generally religious or not.

**Table 1:** Demographic data of the study participants (N = 230)

Data		N	(%)
Gender	Female	159	69,4%
	Male	69	30,1%
	Diverse	0	0,0%
	Not specified	1	0,4%
Marital status	Single	25	10,9%
	Married	157	68,3%
	Divorced	21	9,1%
	Widowed	25	10,9%
	Not specified	2	0,9%
Religion	Yes	165	71,7%
	No	36	15,7%
	Not specified	29	12,6%
Education	No school Leaving Certificate	2	0,9%
	Middle school (until 9th/10th grade)	55	23,9%
	Vocational school	89	38,7%
	High school (until 12th/13th grade)	30	13,0%
	university degree	39	17,0%
	Not specified	15	6,5%

### Information about the participants' illnesses

196 study participants (85.2%) were in the outpatient oncology units because it was their first cancerous disease, 34 patients (14.8%) because of a recurrence. On average, the patients were diagnosed with cancer in the year 2021. 151 participants (65.7%) were currently undergoing treatment at the time of the survey, 79 (34.3%) were in follow-up care.

At 44.7% (N = 101), breast cancer was the most common cancer among the study participants, followed by 25.2% (N = 57) of cancerous haematological diseases, in which for example leukaemia, lymphoma and multiple myeloma were mentioned in the free text field. The third most common type was cancer of the digestive tract (N = 47; 20.8%).

Chemotherapy was administered to 170 patients (74.6%), surgery to 119 patients (52.2%), radiotherapy to 101 patients (44.3%), immunotherapy to 47 patients (20.6%) and hormone therapy to 46 patients (20.2%). Multiple answers were also possible in this part of the questionnaire, because some patients were treated with several forms of therapy (N_answers_ = 494, N_pat_ = 228).

120 participants (52.2%) stated that they had one or more previous illnesses (N_answers_ = 201, N_pat_ = 120). Of these, most reported hypertension (N = 71; 59.2%), type 2 diabetes mellitus (N = 20; 16.7%), osteoarthritis (N = 19; 15.8%) and heart disease (N = 19; 15.8%). Depression was reported in 14.2% of cases (N = 17) and anxiety disorders in 7.5% of cases (N = 9). 73.6% of the pre-existing conditions were treated with medication, 4.7% with psychotherapy and 7.0% with both.

### Evaluation of ASKU

The evaluation of the ASKU shows that the self-efficacy of cancer patients hardly differs from that of the normal population (Beierlein et al. 2014).

The first statement (“I can rely on my own abilities in difficult situations.”) was rated by 221 participants. Most ticked “applies mostly” (50.4%), followed by “applies completely” (29.1%) and “applies somewhat” (10.0%). The assessments of the second (“I am able to solve most problems on my own.”) and third item (“I can usually solve even challenging and complex tasks well.”) were similar. In comparison to the reference values of the ASKU with a mean value of 4.00 and a standard deviation of 0.74 (Beierlein et al.), the results of our questionnaire were nearly the same with a mean value of 3.94 and a standard deviation of 0.82.

### Use of CAM methods

**Fig. 1:**
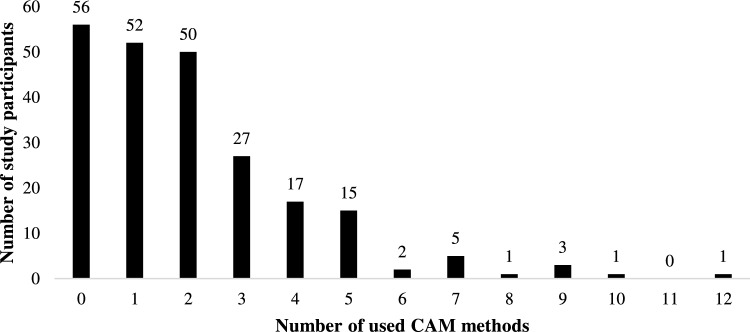
Number of CAM methods used by study participants (N = 230)

The evaluation shows that 174 study participants (75.7%) used some kind of CAM methods (Fig. 1). The other 56 patients (24.3%) did not use any of them. The median value of CAM utilisation was 2.0, the minimum was zero methods per patient and the maximum was twelve methods per patient.

**Fig. 2:**
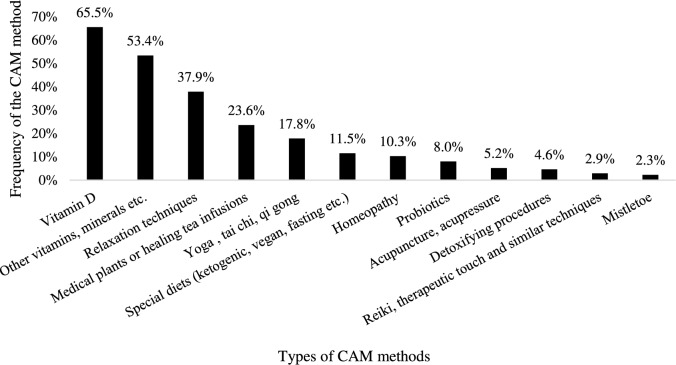
Types of CAM methods used by CAM-using study participants (N = 174)

As summarized in Fig. 2, the patients provided more detailed information on their use of CAM methods. Sports and exercises were not counted as a CAM method in the following analyses and calculations, although it was used by 160 patients, making it the most frequently used method. Therefore, excluding sports, Vitamin D was the most common supplement used by 114 cancer patients (65.5%), followed by other vitamins, food supplements, minerals (N = 93; 53.4%) and relaxation procedures as meditation, mindfulness exercises, autogenic training or progressive muscle relaxation (N = 66; 37.9%) (Fig. 2).

Medical plants, healing tea infusions or secondary plant substances were applied by 41 patients (23.6%). According to the free text field, within this category it was mainly healing tea infusions (N = 14; 34%), curcumin (N = 9; 22.0%), and green teas (N = 8; 19.5%) that were taken. Some study participants were also consuming psyllium husks (N = 2; 4.9%), silica (N = 1; 2.4%), different extracts (e.g. nettle (N = 2; 4.9%) agaricus and coriolus (N = 1; 2.4%), ginkgo (N = 1; 2.4%), dandelion (N = 1; 2.4%), ginger (N = 1; 2.4%), hemp (N = 1; 2.4%), christmas rose (N = 1; 2.4%)), as well as si wu tang (N = 1; 2.4%), ashwagandha (N = 1; 2.4%), artemisinin (N = 1; 2.4%), lycopin (N = 1; 2.4%), broccoli sprouts (N = 1; 2.4%), chaga tea (N = 1; 2.4%), detox tea (N = 1; 2.4%), black cumin oil (N = 1; 2.4%) or ayurvedic herbs (N = 1; 2.4%).

Furthermore, yoga, tai chi, qi gong (N = 31; 17.8%), special diets such as ketogenic, vegan, fasting (N = 20; 11.5%), homeopathy (N = 18; 10.3%), probiotics (N = 14; 8.0%), acupuncture, acupressure (N = 9; 5.2%), detoxifying procedures (N = 8; 4.6%), reiki, therapeutic touch and similar techniques (N = 5; 2.9%) and mistletoe (N = 4; 2.3%) were used (Fig. 2).

### Evaluation of STAI

The trait anxiety part of the questionnaire was filled in by 208 patients. There were people who achieved the minimum trait anxiety score of 20 points and others that achieved the maximum score of 80 points. The mean sum of all trait values added together was 43.52, whereas the comparative value of the calibration sample in the reference population was 35.73 (Laux et al. 1981). Therefore, the level of trait anxiety among the cancer patients was 1.22 times higher than that of the reference population.

The assertions on state anxiety were completed by 206 patients. The mean sum of all state values added together was 44.17, whereas the comparative value of the calibration sample was 37.46 (Laux et al. 1981). Consequently, the level of state anxiety was 1.18 times higher in cancer patients than in the reference population (Grimm 2009).

### Influences on CAM usage

A binomial logistic regression was performed to determine the effect of nine variables (including sex, age, level of education, religion, anxiety as previous illness, depression as previous illness, level of trait anxiety, level of state anxiety and level of self-efficacy) on the application of CAM methods. The binomial logistic regression model was statistically significant, Chi-square (9) = 41.611, p < 0.001, resulting in only a small amount of explained variance (Backhaus et al. 2006), as shown by Nagelkerke's R^2^ = 0.354 (Nagelkerke 1991). Nagelkerke's R^2^ > 0.4 would show a medium effect, Nagelkerke's R^2^ > 0.5 would show a high effect (Backhaus et al. 2006; Nagelkerke 1991). Overall percentage of accuracy in classification was 79.9%, with a sensitivity of 93.1% and a specificity of 27.3%. Of the nine variables included in the regression model, three contributed significantly to predicting CAM usage: gender (p = 0.012), level of education (p = 0.002) and the mean value of the trait anxiety questionnaire (p = 0.024), while the other variables showed no significant effect (Table 2).

**Table 2:** Data from the binary logistic regression analysis showing that gender, level of education and the mean value of the trait anxiety predict CAM usage

		Regression coefficient B	Standard error	Wald	df	Sig	Exp(B)	95% confidence interval for EXP(B)	
								Lower value	Upper value
Step 1^a^	Gender (1)	1,165	,464	6,300	1	,012	3,205	1,291	7,956
	Exact age	,010	,019	,266	1	,606	1,010	,974	1,047
	University degree or high school diploma (1)	3,361	1,087	9,555	1	,002	28,812	3,421	242,671
	Religion (1)	,162	,613	,070	1	,792	1,176	,354	3,908
	Depression (1)	19,565	10,210,826	,000	1	,998	314,057,622,120	,000	
	Anxiety disorder (1)	-,225	1,327	,029	1	,865	,798	,059	10,767
	Mean value of the state items of STAI	−1,454	,749	3,773	1	,052	,234	,054	1,013
	Mean value of the trait items of STAI	1,587	,702	5,114	1	,024	4,889	1,236	19,346
	Mean of all items of ASKU	,153	,335	,210	1	,646	1,166	,605	2,246
	Constant	−1,409	2,201	,410	1	,522	,244		

a. Variables entered in step 1: Mean of all items of the ASKU

The probability of using CAM is 3.2 times higher for women than for men (95%-CI [1.291, 7.956]), 28.8 times higher for patients with a high school diploma or university degree than for patients with a lower level of education (95%-CI [3.421, 242.671]) and 4.9 times higher if the mean value of the trait anxiety part of the questionnaire increases by 1 (95%-CI [1.236, 19.346]). Cohen's f^2^ is 0.55, which according to Cohen (1992) corresponds to a strong effect.

### Correlation of STAI and ASKU

Within the STAI questionnaire, there was a strong positive correlation between the trait anxiety and the state anxiety parts as expected (Pearson: r = 0.834, p < 0.001) (Table 3). The mean value of the trait anxiety correlated moderately negative to the ASKU (Pearson: r = −0.463, p < 0.001), as did the mean value of the state anxiety (Pearson: r = −0.328, p < 0.001).

**Table 3:** Correlations of state anxiety and trait anxiety (STAI) and correlation of state anxiety and trait anxiety each with the self-efficacy scale (ASKU)

		Sum of all state values per person	Sum of all trait values per person
Sum of all trait values per person	Pearson´s Correlation	,834^**^	
	Sig. (2-sided)	< ,001	
	N	202	
Mean of all items of the ASKU	Pearson´s Correlation	–,328^**^	–,463^**^
	Sig. (2-sided)	< ,001	< ,001
	N	201	203

^**^. The correlation is significant at the level of 0.01 (2-sided)

## Discussion

In our survey among cancer patients, the State-Trait-Anxiety-Inventory revealed higher values for both state anxiety and trait anxiety than in the calibration sample of the developers (Laux et al. 1981). Other studies have also observed higher risks for anxiety in oncological patients compared to the normal population (Ghamari et al. 2023; Goerling et al. 2023; Hashemi et al. 2020; Watts et al. 2015, 2014). Therefore, physicians should monitor psychological disorders and changes during cancer treatment to ensure optimal care. According to Naser et al. (2021), antidepressant therapies have so far been used too little in affected patients and mental disorders need to be given more consideration in cancer treatment. However, the diagnosis of cancer is a very challenging situation and often leads to a justified anxiety reaction in a physiological way (Schell et al. 2019). It is therefore important to determine in which patients the anxiety exceeds the physiological level and becomes pathological. This is why empathetic conversations and a trusting physician–patient relationship are important (Zwingmann et al. 2017). Beyond that, oncologists should offer psycho-oncological support to reduce depression and anxiety symptoms in patients (Civilotti et al. 2023; Goerling et al. 2011).

Predictors for the use of CAM methods are higher education (at least high school diploma or university degree) and female gender which was already apparent in other studies (Alsharif 2021; Dufter et al. 2021; Keene et al. 2019; Truant et al. 2013; Verhoef et al. 2005).

In previous surveys in recent years, the number of patients using CAM methods in Europe has been reported to be between 15 to 73% (Molassiotis et al. 2005) depending on the country. In Germany, it ranged from 18% (König et al. 2016) to 80% (Huebner et al. 2014a, 2014b; Männle et al. 2021; Micke et al. 2009; Schönekaes et al. 2003), compared to our current survey results of 76% from various German outpatient oncology centres.

In any case, given this prevalence, physicians and as well medical students need more evidence-based information about CAM, e.g. through lectures or teaching programmes. This enables them to advise patients professionally on CAM, explain possible side effects and risks and avoid overdoses or interactions with conventional therapies (Wolf et al. 2022; McCune et al. 2004). It is also an opportunity to increase patient adherence (Huebner et al. 2014a) if their questions about CAM are taken seriously and discussed together with the physician in form of shared decision making (Hack et al. 2023).

So far, there is few data on how many CAM methods are used by patients. In our study, we showed that in most cases one or two CAM methods per person were used, but there were also cases with up to twelve methods per person. This is important information for physicians to ensure the safety of such patients. The more CAM methods are used, the higher the likelihood of substances interacting with conventional cancer therapies for example via CYP enzymes or P-glycoproteins (Engdal et al. 2009) and causing side effects or physical harm (Wolf et al. 2022). Interaction risks ranging from 65% (Zeller et al. 2013) to even 85% (Loquai et al. 2017) have already been reported. For this reason, oncologists should know about the intake of CAM substances in addition to cancer therapy and prevent vulnerable cancer patients from risks of CAM usage.

The results of the ASKU were similar to previous study results in oncological patients (Festerling et al. 2023; Huebner et al. 2022) and to the reference values of this test in Germany (Beierlein et al. 2014). Accordingly, the self-efficacy of oncological patients compared to the normal population, which was depicted in a random sample, does not appear to differ significantly. Luszczynska et al. (2005) already showed negative correlations between anxiety and self-efficacy. This is consistent with our findings and relevant, as self-efficacy is a personal resource for coping with difficult situations such as cancer (Chirico et al. 2017). As depression and anxiety influence the prognosis of cancer (Wang et al. 2020), physicians should pay particular attention to recognising these disorders and intervening early to improve the outcome and quality of life of patients.

To our knowledge, this is the first study, to assess the association of CAM usage and anxiety. In fact, increased trait anxiety was significantly associated with CAM use. This could be because anxious patients are more worried about the future course of their disease and therefore want to do everything possible to act against the disease themselves (Huebner et al. 2014a). Patients therefore also inform themselves outside of medical facilities via various sources, such as print media, family and friends, internet, social media or non-medical practitioners, about what could help them (Huebner et al. 2014a). Moreover, it is known that trait anxiety influences decision-making (Hartley et al. 2012), which is relevant regarding the application of different CAM methods. Especially in exceptional situations such as a cancer diagnosis, people with an inherently higher level of anxiety are even more insecure about their decisions. For these patients, an informative discussion about their questions and concerns might already help to allay their fears and prevent the use of methods that are associated with higher risk. As anxiety and depression are negatively correlated to resilience (Chuning et al. 2024), it could help those patients to use for example mindfulness-based therapies and resilience interventions that have been proven to reduce the symptoms of anxiety and depression (Chayadi et al. 2022; Ang et al. 2023).

In counselling sessions, knowledge about CAM and the influence of trait anxiety on cancer patients would help oncologists to better assess the needs and mental state of cancer patients and improve their quality of life through appropriate therapies. This can be implemented, for example, through the timely use of psychological or psychotherapeutic support, medication or well-informed usage of complementary and alternative medicine, depending on the individual patient case.

### Limitations

Some questionnaires were not filled in completely. The completed parts of the questionnaire were nevertheless included in the analyses.

In our survey, breast cancer was the most common type of cancer, followed by haematological cancers and cancer of the digestive tract. We tried to include patients with all different types of cancer in the survey by integrating various outpatient oncology centres. However, our study does not accurately reflect the current prevalence of all cancers in the German population. But since the prevalence of cancer entities naturally varies throughout the world and therefore differs from region to region, this is a supply reality.

Patients who do not trust the standard medical treatment may not have been reached by this questionnaire because they are not present in the oncological practices where we distributed the questionnaire. In addition, we only have information from those who were willing to complete the questionnaire. Patients who did not wish to do so may have completely different positive as well as negative attitudes towards CAM methods.

As only the completed questionnaires were returned to us by the oncology centres, we do not know how many patients refused to complete the questionnaire. These could be patients who are not interested in CAM methods. On the other hand, it could also be due to a lack of time or other personal circumstances. Unfortunately, we are unable to track this.

A follow-up survey or second survey of the patients who completed the questionnaire is not possible, as the survey was completely anonymous.

We have not collected any information from hospitalised patients. Their fears and anxiety levels could differ from those of patients in outpatient practices.

As the survey was conducted in Germany and not worldwide, it must be considered when interpreting the results that cultural and ethnic factors (Campesino and Koithan 2010) as well as accessibility to CAM also influence the use of alternative medicine.

The ASKU, an item of the questionnaire considering self-efficacy, was developed for the German-speaking general population aged 18 and over. It is therefore not a special test for cancer patients. However, the ASKU has already been used successfully in some surveys with cancer patients (Festerling et al. 2023; Huebner et al. 2022; Welter et al. 2021).

The results of Nagelkerke's R^2^ show only a small effect of the explained variance. Using other independent variables for the binary logistic regression analysis might have increased the explained variance.

There are many different types of cancer and even within a single cancer entity there are many different sub-characteristics. Depending on the stage of the cancer and the molecular subtype, the therapeutic methods differ enormously (Leitlinienprogramm Onkologie 2021). Therefore, with our sample of 230 patients, it was not possible to analyse specific patterns of therapeutic methods that would indicate CAM users or non-CAM users.

Mental illnesses and anxiety are still very intimate topics for many patients. It can therefore be assumed that there is a higher number of undiagnosed mental disorders. This questionnaire was not intended to create fears that did not previously exist. For this reason, detailed questions about patients' anxiety were not included. However, response bias must be observed. Questions may not have been answered truthfully by study participants in the survey due to social desirability, even though it was anonymous. This was already reported by the developers of the STAI (Laux et al. 1981). A meta-analysis by Ray et al. (2013) nevertheless emphasises the validity of self-report scales.

Some oncological practices that were asked did not take part in the survey or reduced the survey period to a minimum so as not to inconvenience their patients.

## Conclusion

The higher the level of trait anxiety among cancer patients, the more likely it is that CAM methods will be used. Physicians should be aware of this association so that they can better understand the reasons why patients resort to CAM methods. This creates a respectful and empathetic atmosphere to counsel patients on evidence-based CAM methods supporting their needs. In fact, many methods of complementary medicine may enhance patient activation and therefore reduce anxiety. Anxiety should be taken seriously, especially in patients with cancer, and should be diagnosed and treated early to improve quality of life and promote better prognosis. Thus, indirectly well-informed usage of complementary methods as relaxation techniques and other mind–body-methods might be a better and safer recommendation than many alternative substance-bound methods and increase satisfaction and adherence to conventional therapy as well as improve the overall outcome.

## Data Availability

Data is provided within the manuscript or supplementary information files.

## Abbreviations

CAM: Complementary and alternative medicine
ASKU: Self-efficacy short scale
STAI: State-Trait-Anxiety-Inventory
